# Armadillidin H, a Glycine-Rich Peptide from the Terrestrial Crustacean *Armadillidium vulgare*, Displays an Unexpected Wide Antimicrobial Spectrum with Membranolytic Activity

**DOI:** 10.3389/fmicb.2016.01484

**Published:** 2016-09-22

**Authors:** Julien Verdon, Pierre Coutos-Thevenot, Marie-Helene Rodier, Celine Landon, Segolene Depayras, Cyril Noel, Sylvain La Camera, Bouziane Moumen, Pierre Greve, Didier Bouchon, Jean-Marc Berjeaud, Christine Braquart-Varnier

**Affiliations:** ^1^Laboratoire Ecologie et Biologie des Interactions, UMR CNRS 7267, Université de PoitiersPoitiers, France; ^2^Centre de Biophysique Moléculaire, CNRS UPR4301Orléans, France

**Keywords:** antimicrobial peptide, crustacean, glycine-rich peptide, membrane permeabilization, mode of action, scanning electron microscopy, transmission electron microscopy

## Abstract

Antimicrobial peptides (AMPs) are key components of innate immunity and are widespread in nature, from bacteria to vertebrate animals. In crustaceans, there are currently 15 distinct AMP families published so far in the literature, mainly isolated from members of the Decapoda order. Up to now, armadillidin is the sole non-decapod AMP isolated from the haemocytes of *Armadillidium vulgare*, a crustacean isopod. Its first description demonstrated that armadillidin is a linear glycine-rich (47%) cationic peptide with an antimicrobial activity directed toward *Bacillus megaterium*. In the present work, we report identification of armadillidin Q, a variant of armadillidin H (earlier known as armadillidin), from crude haemocyte extracts of *A. vulgare* using LC-MS approach. We demonstrated that both armadillidins displayed broad spectrum antimicrobial activity against several Gram-positive and Gram-negative bacteria, fungi, but were totally inactive against yeasts. Membrane permeabilization assays, only performed with armadillidin H, showed that the peptide is membrane active against bacterial and fungal strains leading to deep changes in cell morphology. This damaging activity visualized by electronic microscopy correlates with a rapid decrease of cell viability leading to highly blebbed cells. In contrast, armadillidin H does not reveal cytotoxicity toward human erythrocytes. Furthermore, no secondary structure could be defined in this study [by circular dichroism (CD) and nuclear magnetic resonance (NMR)] even in a membrane mimicking environment. Therefore, armadillidins represent interesting candidates to gain insight into the biology of glycine-rich AMPs.

## Introduction

By their number and their diversity, arthropods play an essential role in all ecosystems. They are widely distributed and can exploit a very diverse range of niches in both terrestrial and aquatic environments. Thus, their dispersion and survival depend on their defense system implemented to fight against any kind of disease-causing agents and parasites present in their environment. To overcome the impacts of pathogens, arthropods have evolved efficient mechanisms based on two specific and complementary immune responses (Lemaitre and Hoffmann, 2007; Cerenius et al., 2010; Buchon et al., 2014): (i) the cellular response, resulting in phagocytosis of small particles and encapsulation of larger ones and (ii) the humoral response involving the synthesis and the release of several immune proteins into the haemolymph, such as clotting enzymes, phenoloxidase cascade effectors and antimicrobial peptides (AMPs).

After insects, crustaceans are by far the most numerous, diverse and widespread animals in the biosphere. Among them, terrestrial crustacean species are perfectly adapted to the life out of water, notably the terrestrial isopods which constitute an important component of the soil fauna (Jeffery et al., 2010; Hornung, 2011). As they break down more than 10% of the decaying leaf litter of many plant species (Curry, 1987), terrestrial isopods are classified as detritivorous. Thus, they are in permanent contact with abundant and diverse communities of microorganisms (Jeffery et al., 2010).

In all described crustacean species, immune reactions take place in blood (haemolymph) which usually contains three haemocyte types: hyaline, semi-granular and granular (Vazquez et al., 2009; Chevalier et al., 2011). Each haemocyte type is thought to have a primary function, for example, haemocytes containing granules are involved in the production, storage and release of AMPs (Vazquez et al., 2009). In crustaceans, there are 15 distinct AMP families published in the literature which are further clustered into four main groups based on amino-acid composition and structure (Rosa and Barracco, 2010; Smith and Dyrynda, 2015). The groups include (i) Single-domain peptides containing cysteine residues engaged in disulfide bonds such as the anti-LPS factor common to various decapod species (ii) Multi-domain or chimeric AMPs like crustins which are shared by several crustacean species (iii) Unconventional AMPs and (iv) Single-domain linear α-helical AMPs and peptides enriched in specific amino acids.

The great majority of these AMPs were isolated from diverse species of the Decapoda order (shrimp, crab, craysfish, lobster) and only one has been characterized in a terrestrial isopod species. This sole non-decapod AMP has been isolated from the haemocytes of *Armadillidium vulgare* and was named as armadillidin (Herbiniere et al., 2005). Armadillidin is a linear cationic peptide characterized by a high glycine content (47%) and a sixfold repeated motif GGGF(H/N)(R/S). However, the amount of purified armadillidin was insufficient to establish an exhaustive antimicrobial spectrum and to determine its secondary structure (Herbiniere et al., 2005). As we were unable to test many microbial strains, the reported spectrum with only one strain found as sensitive (*B. megaterium*) has to be taken with caution.

In the present work, we report armadillidin H (earlier known as armadillidin) variant peptide named as armadillidin Q identified by LC-MS and isolated from crude haemocyte extracts of *A. vulgare*. In order to provide a deeper characterization of both armadillidins, synthetic peptides were purchased and used to by-passed previous technical barriers. The antimicrobial activity of armadillidin H and armadillidin Q was determined against *B. megaterium* to confirm our previous data, but also against selected Gram-positive and Gram-negative bacteria, yeasts and fungi indicator strains. As both peptides exhibit similar activities, subsequent experiments were only performed with the armadillidin H peptide. Its structure was studied by circular dichroism (CD) and nuclear magnetic resonance (NMR). Morphological changes of bacterial and fungal treated cells were visualized by electronic microscopy. Finally, cell permeabilization assays were performed to determine whether armadillidin H exhibits a membranolytic activity.

## Materials and Methods

### Strains and Culture Conditions

Bacterial and yeast strains used in this study are listed in **Table 1**. Bacteria were grown for 24 h either on nutrient agar plates or broth under shaking (200 rpm), at 28 or 37°C depending on the tested strain. Yeasts were grown for 48 h on Sabouraud agar medium at 37°C. Fungal strains are listed in **Table 2**. *Aspergillus fumigatus* ATCC 16424, *A. terreus* MHR1, and *Scedosporium apiospermum* EC13 were also grown on Sabouraud agar medium, but at 28°C during 5 days. Conidia were then harvested in sterile water, filtered to remove hyphae and adjusted to a working concentration of 10^5^ conidia/ml. *Botrytis cinerea* B05.10 and *Alternaria brassicicola Abra*43 were cultured in 9.5 cm Petri dishes containing Potato Dextrose Agar (PDA, Becton Dickinson) under a 16 h photoperiod at 22°C for 2 weeks to induce sporulation. Then, conidia were harvested in 3 ml of sterile water, filtered through miracloth (EMD Chemicals) and adjusted to a working concentration of 10^5^ conidia/ml.

**Table 1:** Minimum inhibitory concentration (MIC) of both armadillidins against selected microbial strains.

Microbial strains		MIC of armadillidin H (μM)	MIC of armadillidin Q (μM)
Gram-positive bacteria	*Bacillus megaterium* F04	2.37	4.75
	*Bacillus pumilus* NG1	4.75	4.75
	*Bacillus subtilis* LMG 28342	9.5	9.5
	*Micrococcus lysodeikticus* ATCC 4698	2.37	2.37
	*Staphylococcus aureus* ATCC 29213	>19	>19
	*Staphylococcus lentus* 982	4.75	9.5
	*Staphylococcus warneri* RK	>19	>19
Gram-negative bacteria	*Aeromonas hydrophila* LMG 2844	9.5	9.5
	*Enterobacter cloacae* DO3	9.5	9.5
	*Escherichia coli* LMG 2092	9.5	9.5
	*Flavobacterium breve* LMG 4011	9.5	9.5
	*Klebiella pneumoniae* 0502083	19	>19
	*Pseudomonas aeruginosa* PA14	19	>19
	*Pseudomonas fluorescens* MFE01	19	>19
	*Pseudomonas syringae* pv *tomato* DC3000	4.75	9.5
	*Salmonella enterica* J18	19	19
Yeasts	*Candida albicans* ATCC 2091	>19	>19
	*Candida glabrata* ATCC 2001	>19	>19
	*Candida inconspicua* MHR 29	>19	>19
	*Candida parapsilosis* ATCC 22019	>19	>19
	*Candida zeylanoides* CLIB 361	>19	>19

*Bacteria (10^6^ CFU/ml) or blastospores (0.5 × 10^3^ to 2.5 × 10^3^/ml) were incubated with twofold dilutions of a 380 μM peptide stock solution. Results correspond to the MIC after incubation for 24 h (bacteria) or 48 h (yeasts) at 28 or 37°C depending on the tested strain. Results are the mean of three independent experiments. Microbial strains were obtained from various culture collections: ATCC (American Type Culture Collection), CLIB (Centre International de Ressources Microbiennes, CIRM-levures, INRA Micalis, AgroParis tech, France) and LMG (Library of Microbiology Universiteit Gent, Belgium). Other strains were from the laboratory culture collection.*

**Table 2:** Minimum inhibiting concentrations for fungal conidia germination induced by armadillidins.

Fungal strains	MIC^a^ of armadillidin H (HL_50_)^b^ (μM)	MIC^a^ of armadillidin Q (HL_50_)^b^ (μM)
*A. brassicicola Abra*43	7.6 (<1.9)	15.2 (3.8)
*A. fumigatus* ATCC 16424	>38 (>38)	>38 (>38)
*A. terreus* MHR 1	>38 (>38)	>38 (>38)
*B. cinerea* B05.10	>38 (15.2)	>38 (19)
*S. apiospermum* EC13	>38 (<4.7)	>38 (9.5)

*^a^MIC was defined as the lowest concentration of peptide that totally inhibits conidia germination after 36 h. ^b^HL_50_ was defined as the concentration of peptide needed to induce a 50% reduction of hyphae length. Blastospores (0.5 × 103 to 2.5 × 103/ml) were incubated with twofold dilutions of a 380 μM peptide stock solution at 22°C. Results are the mean of three independent experiments. Fungal strains were obtained either from ATCC (American Type Culture Collection) or from the laboratory culture collection. *B. cinerea* B05.10 (Staats and van Kan, 2012) and *A. brassicicola* Abra43 (Joubert et al., 2010) are known reference strains.*

### Synthetic Peptides and Reagents

Synthetic native peptides, armadillidin H (molecular weight of 5259.69 g/mol) and armadillidin Q (molecular weight of 5250.68 g/mol), with the respective amino acid sequences (without C-terminal amidation) GHLGRPYIGGGGGFNRG GGFHRGGGFHRGGGFHSGGGFHRGGGFHSGGSFGYR and GHLGRPYIGGGGGFNRGGGFHRGGGFHRGGGFQSGGGFHRGGGFHSGGSFGYR were purchased from ProteoGenix Corporation (Schiltigheim, France). Stock solutions were prepared in 8% acetonitrile at a final concentration of 2.7 mM and stored at -80°C. Working solutions (380 μM) were prepared by dilution in sterile water. All other reagents were purchased from Sigma–Aldrich (Saint-Louis, MO, USA) unless stated otherwise.

### LC-MS Analysis

The molecular masses of haemocyte crude extracts [prepared as described in (Herbiniere et al., 2005)] and synthetic peptides were determined by electrospray ionization mass spectrometry (ESI-MS) with a Xevo Q-TOF (Waters, Milford, MA, USA) mass spectrometer. Samples were suspended in 50% acetonitrile/0.2% formic acid (v/v). LC-MS mass spectra were performed in positive mode with a cone voltage ramping from 20 to 40 V. The spray voltage was set to 3.0 kV, the source temperature to 120°C and the desolvation temperature to 450°C. The LC separation was conducted on a ProSwift^®^ RP-1S (Dionex) analytical reverse-phase HPLC column (4.6 mm × 50 mm). Separation was carried out using a water/acetonitrile/formic acid 0.2% (v/v) solvent system. After an initial 5 min wash without acetonitrile, elution was achieved at a flow rate of 0.5 ml/min with a 10 min linear gradient from 0 to 40% acetonitrile, followed by a 5 min wash with 80% acetonitrile.

### Bacterial and Yeast Growth Inhibition Assays

Minimum inhibitory concentrations (MIC) of both armadillidins toward various bacterial and yeast strains were measured according to the method detailed elsewhere (Verdon et al., 2008). Yeast suspensions [0.5 × 10^3^ to 2.5 × 10^3^ blastospores/ml in RPMI 1640 medium buffered to pH 7.0 with 0.165 M MOPS (morpholinepropanesulfonic acid)] were incubated with twofold dilutions of a 380 μM armadillidin stock solution. Suspensions were incubated for 48 h at 28 or 37°C depending on the tested strain.

### Filamentous Fungal Growth Inhibition Assays

Hyphal growth inhibition tests were performed in 96-wells plate (Nunc) containing Malt extract (15 g.l^-1^; Duchefa) medium for *B. cinerea* B05.10 and *A. brassicicola Abra*43 or RPMI MOPS medium for *A. fumigatus* ATCC 16424, *A. terreus* MHR1 and *S. apiospermum* EC13 strains. Different concentrations of the working peptide solution were prepared in sterile water. A volume of 5 μl of conidia suspension (10^2^ conidia/μl) was added to the incubation medium containing 50 μl of the peptide solution and 50 μl of culture medium. In parallel, 50 μl of sterile water was added instead of the peptide solution. The microtiter plates were placed on an orbital shaker (30 rpm) and incubated for 36 h at 22°C. Each well was observed under an inverted microscope (LEICA, DMI 6000B) and the length of three hyphae (hl) was measured on three independent replicates. Length averages were calculated and the percentage of inhibition was determined according to the following formula: % inhibition = (100-(100/hl_control_) × hl_peptide_).

### Bactericidal Activity Assays

*Pseudomonas syringae* DC3000 or *B. megaterium* F04 cells were grown to an OD_600_ between 0.15 and 0.35. Bacteria were then appropriately diluted in 10 mM sodium phosphate buffer (pH 6.8) to 10^6^ CFU/ml. Serial twofold dilutions of armadillidin H were achieved in sterile 10 mM sodium phosphate buffer (pH 6.8) and added (15 μl) to bacterial suspension (285 μl) at a starting concentration of 4.75 μM. Suspensions were incubated for 1 h at 28 or 37°C depending on the tested strain. Controls were run without peptide (only with the peptide solvent containing acetonitrile). The number of colony-forming units (CFU) was determined by plating 10-fold serial dilutions of bacterial suspensions on NB agar plates after 24 h of incubation at 37°C for *B. megaterium* or 36 h at 28 °C for *P. syringae*.

### Time-Killing Assays

Exponentially growing bacteria in NB (*B. megaterium* F04 and *P. syringae* DC3000) were appropriately diluted in 10 mM sodium phosphate buffer (pH 6.8) to obtain a concentration of 10^6^ CFU/ml. Aliquots of 950 μl were incubated (37°C for *B. megaterium* and 28°C for *P. syringae*) with 50 μl of 10 mM sodium phosphate buffer (pH 6.8) containing armadillidin H at a final concentration of 1.2 μM for *P. syringae* and 4.75 μM for *B. megaterium*. Controls were run without peptide. At different times, suspensions were diluted in 10 mM sodium phosphate buffer (pH 6.8) and spread onto NB agar plates. After an overnight incubation at 37°C for *B. megaterium* or 36 h at 28°C for *P. syringae*, CFU were counted.

### Scanning (SEM) and Transmission (TEM) Electron Microscopy

Exponentially growing bacteria in NB (*B. megaterium* F04 and *P. syringae* DC3000) were appropriately diluted in 10 mM sodium phosphate buffer (pH 6.8) to obtain a concentration of 5.10^7^ CFU/ml. Bacteria were then treated for 15 min at 28°C (*P. syringae* DC3000) or 37°C (*B. megaterium* F04) with armadillidin H concentrations that induce the highest loss of cultivability (determined by counting CFU as described above for bactericidal activity assays) and that allow the presence of a pellet to work on: 9.5 μM for *P. syringae* and 2.37 μM for *B. megaterium*. *A. brassicicola Abra*43 mycelium was collected after 24 h of culture at 22°C in absence or in presence of 7.6 μM armadillidin H, centrifuged (5000 × *g*, 10 min) and fixed according to the following procedure. Cells were fixed for 1 h with 2.5% glutaraldehyde in 1 M phosphate buffer, pH 7.1. After PBS washes, bacterial cells were post-fixed for 45 min in 1% osmium tetraoxyde in phosphate buffer. Dehydration was carried out using successive incubations of increasing ethanol concentrations (from 70 to 100%). Cells were then resuspended in 100% ethanol and separated for SEM or TEM. Each part was centrifuged at 10,000 rpm for 10 min. For TEM, the *P. syringae* DC3000 or *B. megaterium* F04 pellet was included in epon resin and after 24 h of polymerisation,70 nm sections were cut using an ultramicrotome UC6 (Leica). Uranyl acetate (2% in 70% ethanol) and lead citrate were used as contrasting agents for electron microscopy (JEOL 1010 à 80 KV). TEM on cells was recorded using Olympus digital camera Quemesa with Item software. For SEM, bacterial cells were deposited on a 12 mm cover glass and dried by HMDS treatment (hexamethyldisilazane). The surface of the coverglass was sputter-coated in a vacuum with an electrically conductive 25 nm thick layer of gold–palladium alloy coating system (BALTEC Scd 005 sputter coater). SEM images were then recorded with a scanning electron microscope (JEOL 840) at 15 kV.

### Membrane Permeabilization Assays

*Pseudomonas syringae* DC3000 or *B. megaterium* F04 cells were grown to an OD_600_ between 0.15 and 0.35. Bacteria were centrifuged (10000 × *g*, 30 s) and pellets were suspended in 10 mM sodium phosphate buffer (pH 6.8) to approximately 5.10^7^ CFU/ml. Bacterial suspensions were treated with armadillidin H at a final concentration of 9.5 μM for *P. syringae* and 2.37 μM for *B. megaterium*. Suspensions were incubated for 15 min at 28 or 37°C depending on the tested strain. Controls were run without peptide and with 0.1% Triton X-100 for maximal lysis activity. One half of the cell suspensions was analyzed using epifluorescence microscopy (Olympus BX41) after 15 min of bacterial staining with 5 μM SYTO9 (S9) and 7 μM Propidium Iodide (PI) from the Live/Dead BacLight kit L-7005 (Invitrogen, Cergy Pontoise, France), as recommended by the supplier. Damaged bacteria (PI positive) and total bacteria (S9 positive) were counted in each observed field for a total of 10 fields/slide and three slides/condition. Results were expressed as permeabilization: % permeabilization = (number of PI^+^ bacteria/total number of bacteria) × 100. The other half of the bacterial suspensions was appropriately diluted in 10 mM sodium phosphate buffer (pH 6.8) and spread on NB agar plates. After an overnight incubation at 37°C for *B. megaterium* or 36 h at 28°C for *P. syringae*, CFU were counted.

For *A. brassicicola Abra*43, the membrane permeabilization assay was adapted from Joubert et al. (2010). Briefly, 24 h-old germinating conidia grown on malt extract were exposed to 7.6 μM armadillidin H or 0.1% Triton X-100 as positive control for 4 h. After treatment, SYTOX Green was added to the incubation mixture (final concentration of 2 μM) as indicated by the supplier and samples were observed using epifluorescence microscopy as described above.

### Hemolytic Activity Assay

Hemolytic activity of armadillidin H was measured by detection of released hemoglobin from fresh human erythrocytes. Prior to the assay, the blood was centrifuged (2000 × *g*, 3 min, 4°C) to collect erythrocytes. Erythrocytes were washed three times with PBS and adjusted to a final concentration of 10% (∼1.10^8^ cells/ml as determined by blood cells counting with a hemocytometer). Reactions were performed in 1 ml mixtures containing 1% erythrocytes (v/v) and variable amount of peptides or an equivalent volume of PBS buffer. Serial twofold dilutions of peptide were performed in PBS buffer and added to 1% human erythrocytes at a starting concentration of 19 μM. Samples were incubated at 37°C for 30 min. Erythrocytes were removed by centrifugation (2000 × *g*, 3 min, 4°C) and the absorbance of the supernatant was measured at 576 nm. The level of hemolysis when suspending erythrocytes in 0.1% Triton X-100 was considered 100%. Three experiments were carried out in duplicates.

Human blood was obtained from the French national blood transfusion organization, “Etablissement Français du Sang - Centre Atlantique,” under the agreement number CA-PLER-2014 089. Donors gave their written and informed consent for the use of blood samples in research.

### Circular Dichroism (CD) Analysis

Circular dichroism spectra were recorded on a JASCO V-670 spectrometer in the range 190–260 nm, every 0.5 nm (cell length 1 cm), with an armadillidin H sample of 19 μM in 100 mM phosphate buffer, pH 6.0. Then, 50% TFE were added. For spectral deconvolution, the three computer programs Selcon3, CDsstr, and CONTIN/LL have been used, available in the CDPro software package (Sreerama and Woody, 2000) or used at the internet-based CD analysis site DICHROWEB (Whitmore and Wallace, 2008), with selected bases corresponding to soluble proteins (either structured or denaturated).

### Nuclear Magnetic Resonance (NMR) Spectroscopy Analysis

Nuclear magnetic resonance spectra were recorded on an advance III HD BRUKER 700 MHz spectrometer equipped with a TCI cryoprobe, and were processed with the NMRPipe/NMRDraw software package (Delaglio et al., 1995). A set of 2D ^1^H NOESY (160 ms), 2D ^1^H TOCSY (80 ms), natural abundancy ^13^C HSQC were performed at 298 K on a 0.1 mM (300 μl in a 3 mm tube) solution of armadillidin H in 100 mM phosphate buffer, pH 5.2, and 10% of D_2_O ^1^H chemical shifts were referenced to the DSS signal at 0 ppm. After lyophilisation, the same sample was solubilized in H_2_O/TFE (50/50, v/v) to acquire 160 ms NOESY, 80 ms TOCSY experiments, and ^13^C HSQC with the previously defined parameters.

## Results

### Discovery of an Armadillidin H Variant in Haemocytes of *Armadillidium vulgare*

**FIGURE 1:**
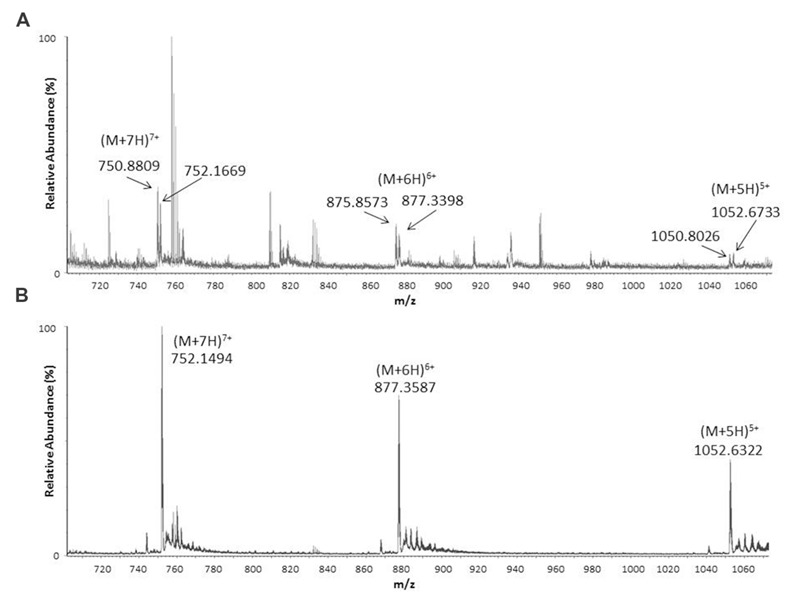
**Electrospray ionization mass spectrometry (ESI-MS) spectra showing pseudo-molecular multicharged ions obtained in positive mode from crude *Armadillidium vulgare* haemocytes extract **(A)** and synthetic armadillidin H (B)**.

To find out if armadillidin is the only AMP produced and stored in *A. vulgare* haemocytes (Herbiniere et al., 2005), a crude extract prepared from haemocytes of 100 animals was analyzed by LC-MS. A weak peak was observed at 9.3 min corresponding to the retention time found for synthetic armadillidin (not shown). The corresponding spectrum (**Figure 1A**) showed the same multicharged ions as those observed for the analysis of synthetic armadillidin (**Figure 1B**). As expected, the calculated monoisotopic molecular mass was 5256.15 Da. Surprisingly, a second set of multicharged ions was observed, close to the armadillidin peaks. The monoisotopic molecular mass calculated for this second peptide was 5247.13 Da. We hypothesized that the difference of 9 Da between these two peptides could correspond to the replacement of a His (H) residue by a Gln (Q) or a Lys (K) residue. To verify this hypothesis, an *A. vulgare* transcriptome generated by another research program (ANR-10-BLAN-1701 ImmunSymbArt; Sequence Read Archive SRA database accession numbers: SRX564995-SRX565004) and, available in the laboratory, was screened using the blastx alignment tool^1^. Results showed the presence of two variants of armadillidin encoded in the genome of *A. vulgare*, the second one differing from the previously described one (Herbiniere et al., 2005) by only one amino acid substitution: a H is replaced by a Q at position 33. Therefore, we named this variant armadillidin Q and the first discovered armadillidin was renamed armadillidin H.

### Antimicrobial Activity of Both Armadillidins

In order to determine the putative sensitivity of various microbial strains to both armadillidins, an antibacterial activity assay was performed and the MIC was determined for each tested strain. MIC was defined as the lowest concentration of peptide that totally inhibits the visible growth of a selected bacterial or yeast strain after a chosen incubation period (24 h for bacteria and 48 h for yeasts). Results are given in **Table 1**. Armadillidin H was shown to be active against all the tested bacteria, excepted for two staphylococcal strains (**Table 1**). Among sensitive strains, four appeared to be highly susceptible as they were inhibited at low peptide concentrations (∼2–5 μM). Similar results were obtained for armadillidin Q as determined MICs were in the same range as for the parent peptide (**Table 1**). However, concerning yeasts, no growth inhibition has been measured for both peptides (**Table 1**). All together, these results indicated that both armadillidins displays a similar broad antibacterial spectrum.

When tested against *A. brassicicola Abra*43, both peptides displayed a high inhibitory effect on mycelium growth. Indeed, the growth was completely stopped at 7.6 μM for armadillidin H and at 15.2 μM for armadillidin Q (**Table 2**) and conidial germination never occurred even after 2 weeks of culture (data not shown). The difference in the MIC of the two peptides against *A. brassicicola Abra*43 corresponds to only one dilution. Because we did not test intermediary dilutions, MIC for armadillidin Q could be close to 8 μM thus not significantly different. For *B. cinerea* B05.10, conidial germination still occurred when treated at the highest tested peptides concentration (38 μM; **Table 2**), indicating that *B. cinerea* B05.10 is less sensitive to armadillidins than *A. brassicicola Abra*43. However, a 50% hyphae length reduction was observed at 15.2 μM for armadillidin H and 19 μM for armadillidin Q (**Table 2**). Similar results were obtained for *S. apiospermum* EC13 with a 50% hyphae length reduction observed at 4.7 μM for armadillidin H and 9.5 μM for armadillidin Q (**Table 2**). No effect was observed on the mycelium length for the two others fungi, *A. fumigatus* ATCC 16424 and *A. terreus* MHR1, over the tested concentration range of both armadillidins.

As both peptides displayed the same inhibition spectrum with close MICs values, we decided to use only armadillidin H for the subsequent biological tests and the structure determination. Moreover, *B. megaterium* F04, *P. syringae* DC3000, and *A. brassisicola Abra*43 were chosen as models to study the mode of action of armadillidin H as they were representative of sensitive Gram-positive, Gram-negative and fungi, respectively. In addition, these microorganisms are known to be relatively abundant and ubiquitous in soil where *A. vulgare* is living (Fierer et al., 2012).

### Bactericidal Activity of Armadillidin H and Killing Kinetics

**FIGURE 2:**
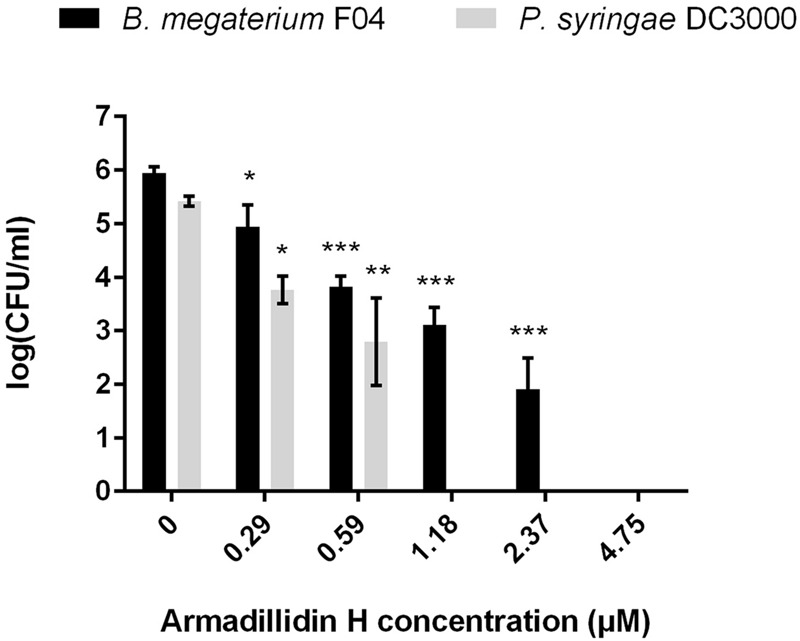
**Bactericidal effect of armadillidin H toward selected bacteria.** Bacteria were diluted to 10^6^ CFU/ml in 10 mM sodium phosphate buffer (pH 6.8) and incubated with various concentrations of peptides for 1 h at 28 or 37°C depending on te tested strain. Controls were run without peptide. Three experiments were carried out in duplicates and the results are expressed as means. Bar scales indicate standard deviation. As the sample data reach the assumptions (Shapiro–Wilk tests for normality and Bartlett tests for the homogeneity of variances), one-way analyses of variance (ANOVA) followed by *post hoc* tests (Tukey HSD) have been performed; CFU for each concentration was compared with control: ^∗^*p* < 0.05, ^∗∗^*p* < 0.01, ^∗∗∗^*p* < 0.001.

**FIGURE 3:**
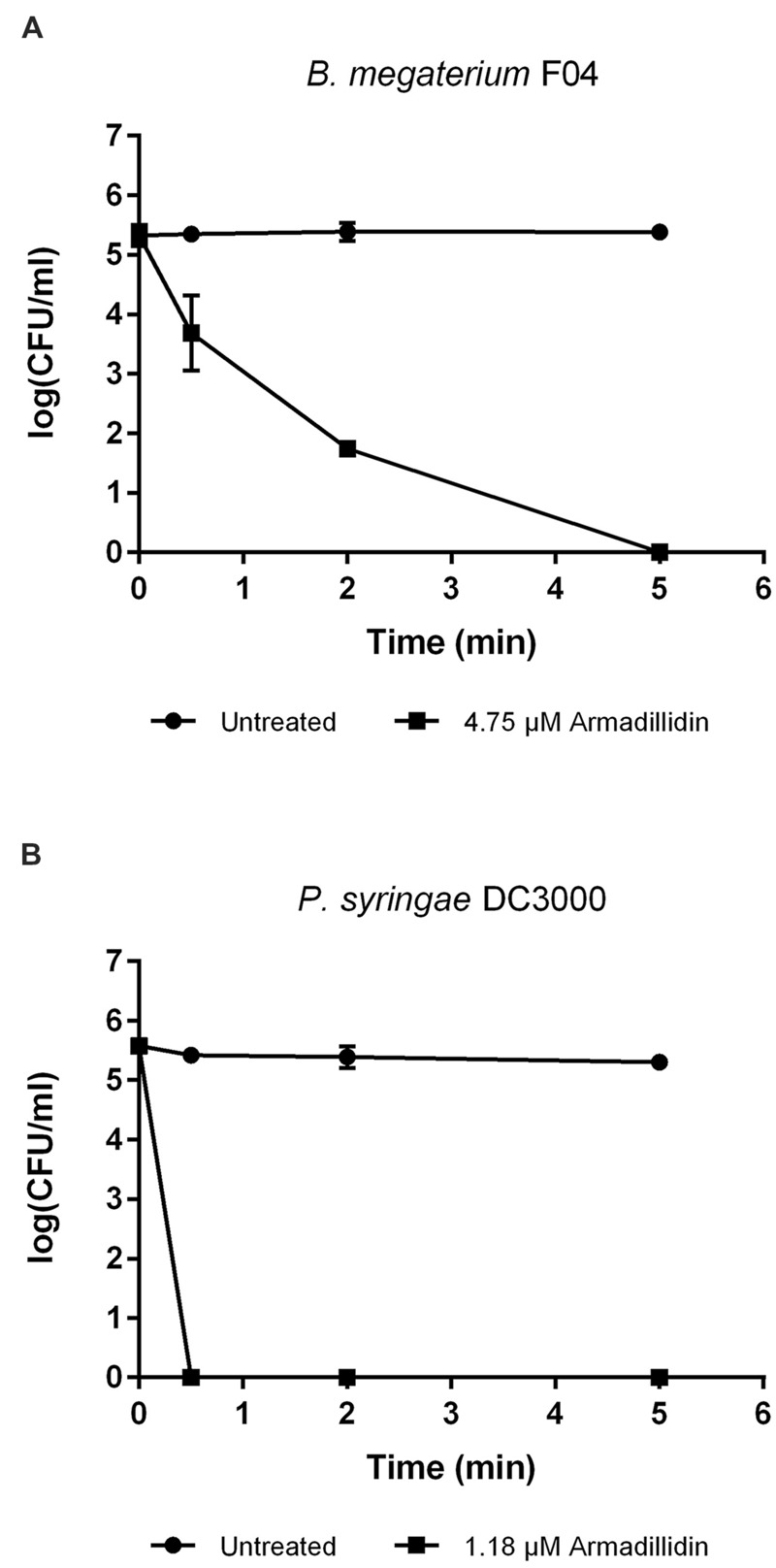
**Time-killing curves of selected bacteria by armadillidin H. **(A)***B. megaterium* F04 and **(B)***P. syringae* DC3000.** Bacteria were diluted to 10^6^ CFU/ml in 10 mM sodium phosphate buffer (pH 6.8) and incubated 5 min with armadillidin H. Controls correspond to bacteria incubated in buffer without peptide. Two experiments were carried out in triplicates and the results are expressed as means. Bar scales indicate standard deviation.

To verify whether armadillidin H has the capacity to kill bacteria or to only inhibit their growth, *B. megaterium* F04 and *P. syringae* DC3000 were incubated for a duration of 1 h in the presence or absence of the peptide. After treatments, serial dilutions were spread on NB agar plates so as to quantify viable bacteria. As shown in **Figure 2**, a decrease of CFU was obtained while increasing armadillidin H concentration, in a dose-dependent manner. No bacterial growth was observed at 4.75 μM for *B. megaterium* F04 and 1.18 μM for *P. syringae* DC3000 indicating that armadillidin H acts as a bactericidal peptide. Moreover, the time-killing curves revealed that armadillidin H induced the killing of all the *B. megaterium* population within 5 min (**Figure 3A**). By contrast, the killing of all the *P. syringae* population was achieved faster as no CFU was detected after a 30 s incubation period with armadillidin H (**Figure 3B**). These results indicated that the bactericidal effect of armadillidin H occurs very quickly on both Gram-positive and Gram-negative bacteria.

### Morphological Changes Induced by Armadillidin H on Bacterial and Fungal Cells

**FIGURE 4:**
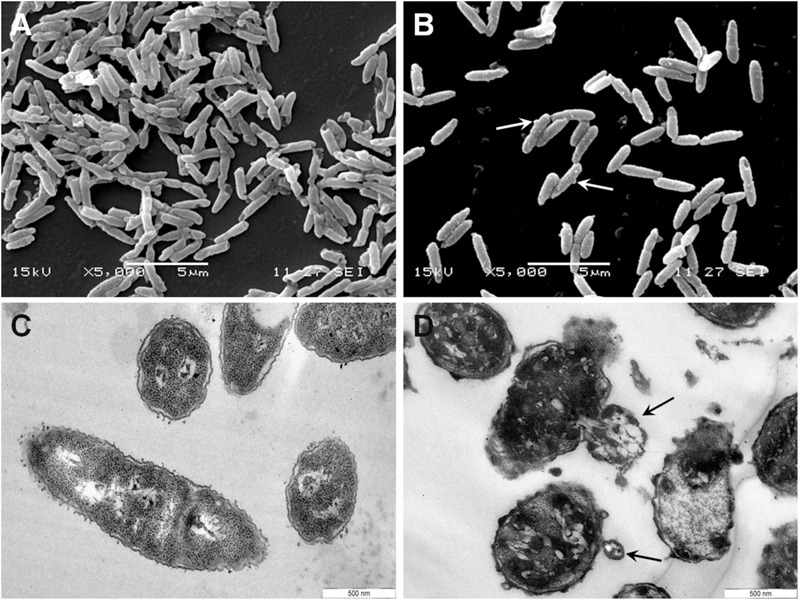
**Scanning and transmission electron micrographs of armadillidin H treated *P. syringae* DC3000.** Cells (5.10^7^ CFU/ml) were incubated at 28°C for 15 min without armadillidin H **(A,C)** or with 9.5 μM armadillidin H **(B,D)** and samples were analyzed by SEM **(A,B)** and TEM **(C,D)**. Blebs on cell surface are indicated by arrows.

**FIGURE 5:**
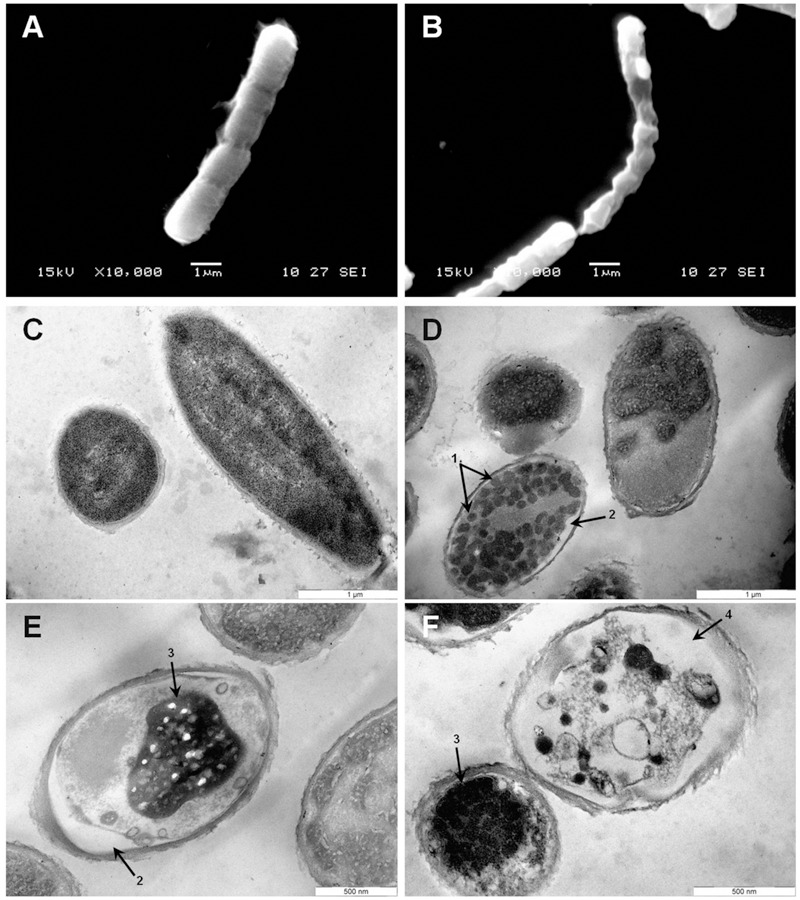
**Scanning and transmission electron micrographs of armadillidin H treated *B. megaterium* F04.** Cells (5.10^7^ CFU/ml) were incubated at 37°C for 15 min without armadillidin H **(A,C)** or with 2.37 μM armadillidin H **(B,D–F)** and samples were analyzed by SEM **(A,B)** and TEM **(C–F)**. Arrows: (1) Electron dense dots; (2) Membrane detachment; (3) Highly condensed electron dense region; and (4) large electron-lucent area.

**FIGURE 6:**
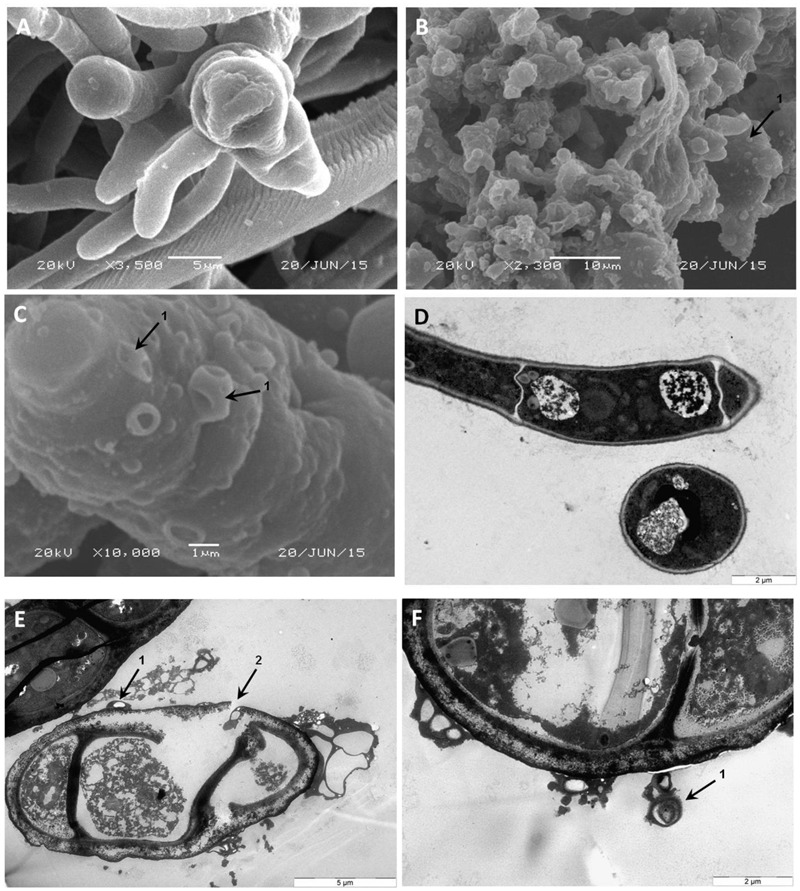
**Scanning and transmission electron micrographs of armadillidin H treated *A. brassicicola Abra*43 mycelium structure.** Conidia were cultured in malt extract culture medium without armadillidin H **(A,D)** or supplemented with 7.6 μM armadillidin H **(B,C,E,F)**. After 24 h of treatment at 22°C, mycelium was fixed and analyzed by SEM **(A–C)** and TEM **(D–F)**. Arrows: (1) vesicle-like structures; and (2) rupture of the mycelium cell wall integrity.

To gain insight into the direct effect of armadillidin H on the morphology of treated cells, we used SEM and TEM approaches. Representative electron micrographs of selected experiments are shown in **Figures 4–6** for *P. syringae, B. megaterium*, and *A. brassicicola*, respectively. Control *P. syringae* cells had a smooth and normal surface morphology (**Figures 4A,C**). However, incubation with 9.5 μM armadillidin H showed severe surface damages like membrane wrinkling and bubbling (**Figures 4B,D**). Moreover, many cells had lost their intracellular organization and membrane integrity. Regarding *B. megaterium*, untreated samples consisted mainly of single cells or diplobacilli displaying an intact smooth surface (**Figure 5A**). TEM analysis also revealed intact cell walls and cytoplasmic membranes (**Figure 5C**). The armadillidin H treatment greatly affected the cell morphology of *B. megaterium* with a deep roughening of the cell surface (**Figure 5B**) and a considerable loss of the typical subcellular organization with different steps in peptide-mediated killing (**Figure 5D**). Indeed, it began with the condensation of cytoplasmic material and detachment of the cell membrane (**Figure 5D**). In the next step, this condensation evolved to a highly electron-dense amorphous region (**Figures 5D,E**). Some alterations of the cell wall were also noticed. Finally, cells adopted a ghost-like appearance highlighted by the presence of a large electron-lucent area (**Figure 5F**). The cells seemed to have lost a part of their cytoplasmic contents, although the overall cell shape was still recognizable. Concerning mycelium of *A. brassicicola*, in the control condition, it displayed a smooth surface and numerous ramifications (**Figure 6A**). In contrast, mycelium of *A. brassicicola* treated with 7.6 μM armadillidin H showed a complete disruption of branching and an uncontrolled cell proliferation (**Figure 6B**). At higher magnification, vesicle-like structures protruding from the mycelium cell wall were observed, leading to a granular and irregular surface (**Figure 6C**). TEM observations confirm these findings (**Figures 6D–F**). Indeed, untreated mycelium (**Figure 6D**) showed cells with smooth surface and cell-wall, the plasma membrane is also clearly defined. The cytoplasm is well-structured and dense with visible organelles and endoplasmic reticulum. By contrast, fungal cells treated with armadillidin H (**Figures 6E,F**) have lost their regular shape. We observed extracellular blebs, already visible in SEM. The cell-wall is rough and the cytoplasm structure is clearly altered. Plasmalemma and organelles appear to be degraded showing that cells are probably collapsed.

### Armadillidin H Induced Membrane Permeabilization

**FIGURE 7:**
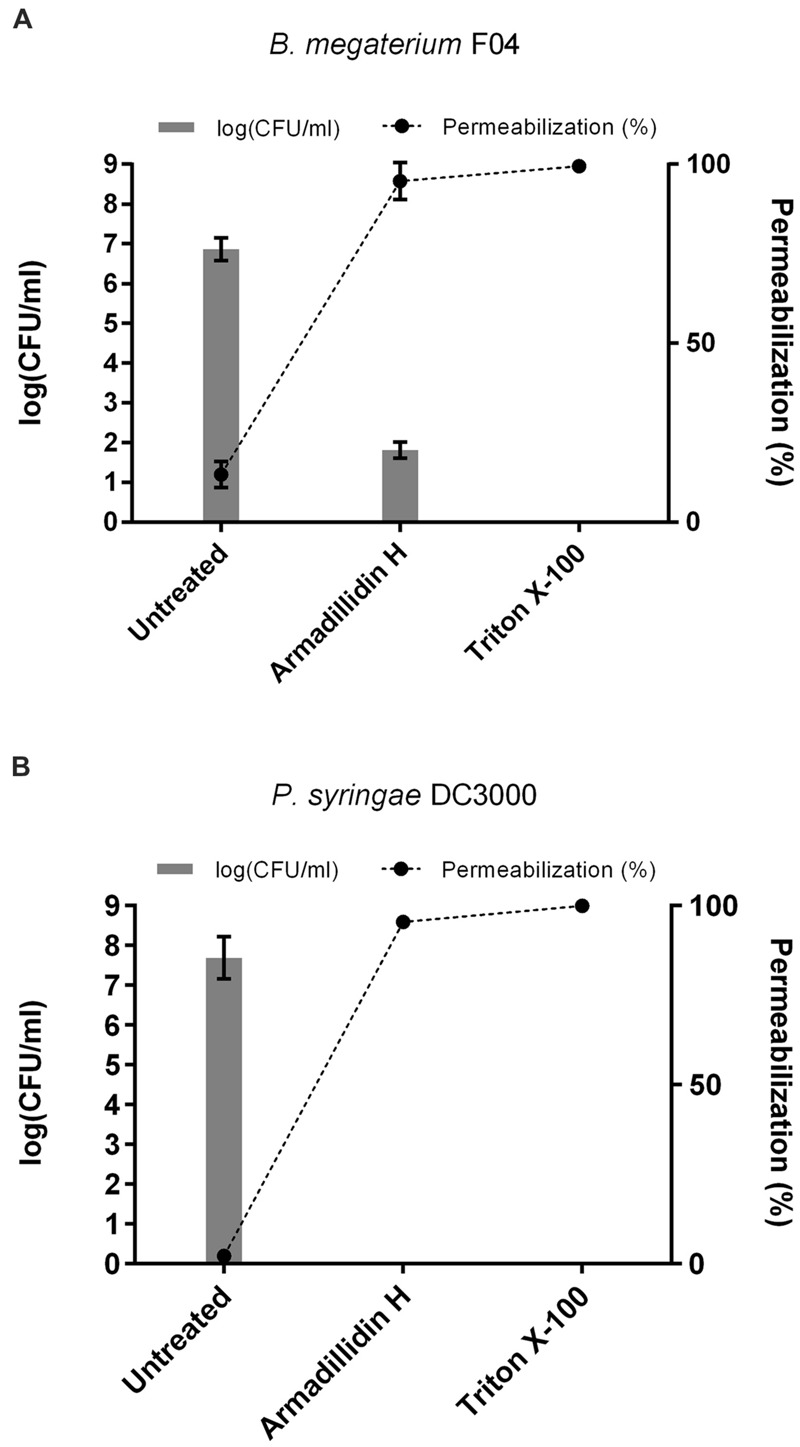
**Bacterial membrane permeabilization induced by armadillidin H. (A)** *B. megaterium* F04 and **(B)** *P. syringae* DC3000. Bacteria (5.10^7^ CFU/ml) were treated with armadillidin H and incubated for 15 min at 28 or 37°C depending on the tested strain. Controls were run without peptide and with 0.1% Triton X-100 for maximal lysis activity. Results of permeabilizaton were expressed as (% permeabilization) = (number of IP^+^ bacteria/total number of bacteria) × 100 and bacterial viability was expressed as log(CFU/ml). Three experiments were carried out in triplicates and the results are expressed as means. Bar scales indicate standard deviation.

**FIGURE 8:**
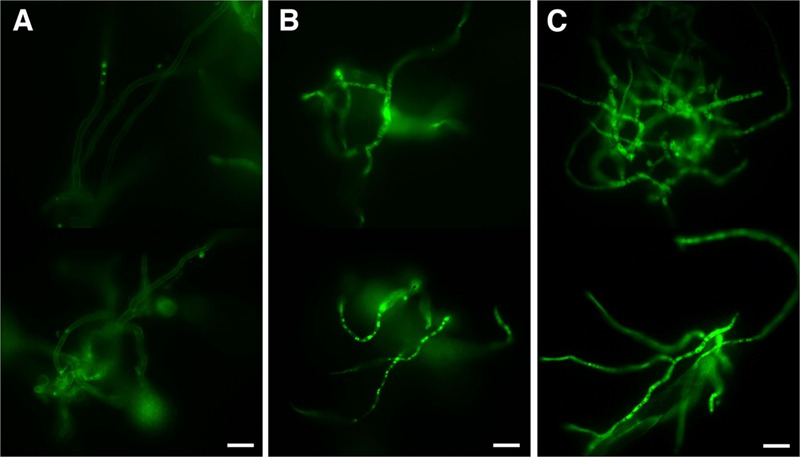
**Fungal membrane permeabilization induced by armadillidin H.** Membrane integrity was tested using SYTOX green uptake assays on 24 h germinated conidia treated for 4 h with peptide solvent **(A)**, 7.6 μM armadillidin H **(B)**, and 0.1% Triton X-100 **(C)**. Scale bars = 20 μm.

As the observed ultrastructure of armadillidin H-treated cells strongly suggested a membranolytic step in the mode of action of this peptide, we investigated the membrane permeabilization potency of this peptide using propidium iodide that easily penetrates cells with compromised membranes. In parallel, the viability of bacteria was assayed by CFU counts so as to potentially correlate cell permeabilization and bacterial death (**Figure 7**). In the case of *B. megaterium*, 2.37 μM armadillidin H induced 95.3% (±5.1%) permeabilization of the cell population and a 5 log-reduction of the CFU number (from 6.9 ± 0.3 to 1.8 ± 0.2; **Figure 7A**). Concerning *P. syringae*, 9.5 μM armadillidin H induced 95.4% (±1.1%) permeabilization of the cell population while no CFU growth was observed (from 7.7 ± 0.5 to 0; **Figure 7B**). Thus, armadillidin H induced permeabilization of the bacterial membrane, thereby provoking, directly or not, cell death. Concerning the membrane integrity of *A. brassicicola*, as seen in **Figure 8**, very little intracellular green fluorescence (SYTOX Green) was detected in untreated hyphae (**Figure 8A**). By contrast, many intracellular green areas were observed after 4 h of exposure with 7.6 μM armadillidin H resulting from dye uptake (**Figure 8B**). Similar results were observed when hyphae were incubated with 0.1% Triton X-100 as a control (**Figure 8C**). Taken together, these results indicate that armadillidin H permeabilizes *A. brassicicola* hyphae.

### Hemolytic Activity of Armadillidin H

**FIGURE 9:**
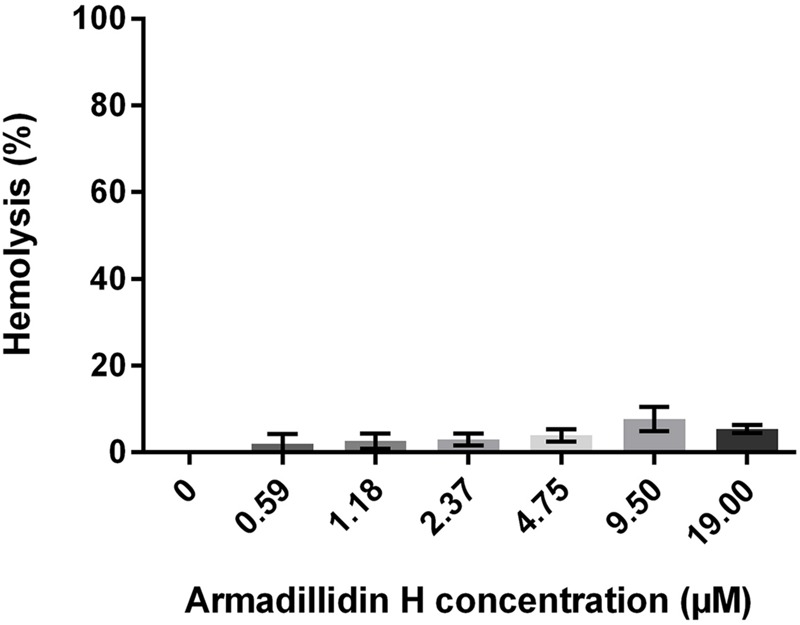
**Hemolytic activity of armadillidin H toward human erythrocytes.** Erythrocytes were diluted to 10^7^ cells/ml in PBS buffer and incubated with various concentrations of armadillidin H for 30 min at 37°C. Controls were run with 0.1% Triton X-100 for maximal hemolysis activity. Three experiments were carried out in triplicates and the results are expressed as means. Bar scales indicate standard deviation.

To investigate whether armadillidin H has any effect on mammalian membranes, its hemolytic potency was assayed. After incubating fresh human erythrocytes with the peptide up to 19 μM concentrations, no hemoglobin release was observed, indicating that armadillidin H does not cause lysis of erythrocyte membrane (**Figure 9**).

### Structure of Armadillidin H

Preliminary to the experimental structural evaluation, the sequence of armadillidin H was submitted to MedoR (MEtaserver of DisORder), a metaserver for predicting protein disorder (Lieutaud et al., 2008). Whatever the predictor questioned by the MedoR metaserver, no secondary structure could be predicted. The FoldIndex software (Prilusky et al., 2005) augurs armadillidin H to be unstructured in the range 1–53, i.e., from the first to the last residue. The two CD spectra of armadillidin H recorded in 100 mM phosphate buffer pH 6.0, with or without the addition of 50% TFE display the same shape. When a deconvolution program, whatever the bases of soluble proteins chosen – structured or not – is applied, a large majority of residues are predicted to be unstructured or in turn, with only 25–30% of residues which could be in an extended conformation. However, the prediction of β-sheet by CD is generally trickier than the prediction of helices. Due to the dilution by a factor of two, the CD spectrum with TFE is less intense, but totally superimposable with the spectra recorded without TFE (Supplementary Figure S1), which means that the peptide does not show any propensity to form secondary structures when the media mimics membrane environment.

**FIGURE 10:**
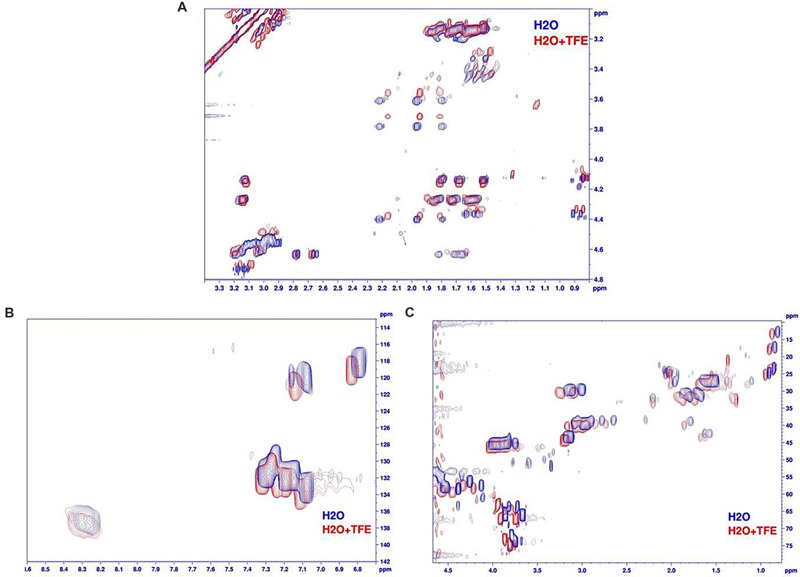
**Comparison of armadillidin H NMR spectra recorded in H_2_O/D_2_O (phosphate buffer pH 5.2, blue spectra) and with 50%TFE (red spectra). (A)** Superimposition of the aliphatic regions of TOCSY spectra (Tm 80 ms), **(B)** superimposition of the aromatic region of natural abundancy ^13^C HSQC spectra and **(C)** superimposition of the aliphatic regions of natural abundancy ^13^C HSQC spectra.

The NMR spectra showed a weak dispersion of 1H chemical shifts in the amide region (between 7.7 and 8.5 ppm), indicative of an unstructured peptide. Moreover, Hα and Cα chemical shifts, known to be particularly sensitive to secondary structures, are restricted. Finally, the superimposition of TOCSY and NOESY spectra (data not shown) does not highlight any additional peaks in the NOESY spectra that could be indicative of medium or long range NOE connectivities typical of 3D structuration. Then armadillidin H is not structured in our experimental conditions (0.1 mM in phosphate buffer pH 5.2). The addition of 50% of TFE (**Figure 10**) does not lead in significant shifts (except for the NH protons sensitive to “pH” variations induced by the addition of TFE). Hα and Cα chemical shifts, sensors of secondary structure variations, are not influenced by the addition of TFE. This means that the peptide remains unstructured, even in presence of TFE 50%, then it does not show any propensity to form secondary structures when the media mimics membrane environment.

## Discussion

Armadillidin H is a crustacean linear cationic glycine-rich peptide with a remarkable amino acid composition as seen in other glycine-rich AMPs (Baumann et al., 2010). Indeed, only ten amino acids make up the peptide which is mainly composed of G (47.2%), F (13.2%), H (11.3%), and N (11.3%) (Herbiniere et al., 2005). Another striking feature of this peptide is the presence of a sixfold repeated motif GGGF(H/N)(R/S) representing 68% of the sequence. Nowadays, there are 15 distinct AMP families described so far in crustaceans (Rosa and Barracco, 2010). Most of them are characterized by a high diversity of isoforms and it is proposed that this multiplicity could confer a broad spectrum of activity to a single peptide family (Rosa and Barracco, 2010; Smith and Dyrynda, 2015). Firstly, to determine whether armadillidin H is the only isoform produced and stored within *A. vulgare* haemocytes, a LC-MS analysis was conducted using a crude extract of haemocytes. Results showed the presence of an extra peptide with a calculated average molecular weight of 5250.68 Da, 9 Da lower than the molecular weight of armadillidin H (Herbiniere et al., 2005). Results were confirmed by the presence of the corresponding cDNAs in *A. vulgare* RNAseq libraries. Hence, *A. vulgare* expresses two armadillidin peptides, the previously identified armadillidin H and a second isoform, named armadillidin Q. Secondly, we showed that both chemically synthesized armadillidins have equivalent antimicrobial activity against Gram-positive bacteria, Gram-negative bacteria and filamentous fungi since MICs values for both peptides are all within a twofold difference. Moreover, the lowest MICs values were mainly measured for Gram-positive bacteria and *P. syringae* DC3000. These values are in the same micromolar range than MICs values obtained for efficient antibacterial compounds already described in literature (Verdon et al., 2008; Loiseau et al., 2015). Surprisingly, no activity was detected against yeasts of the *Candida* genus. Indeed, glycine-rich peptides are generally reported to be active against Gram-negative bacteria and/or fungi (including yeasts) as it was described for arthropod AMPs like acanthoscurrins (Lorenzini et al., 2003), coleoptericin (Bulet et al., 1991), ctenidins (Baumann et al., 2010), diptericin A (Keppi et al., 1989), holotricin 3 (Lee et al., 1995), or tenecin 3 (Lee et al., 1996), frog AMPs like leptoglycin (Sousa et al., 2009) and plant AMPs like shepherins (Park et al., 2000) (**Table 3**). However, some exceptions exist like hyastatin from the spider crab *Hyas araneus* that exhibits a broad spectrum of antimicrobial activity (Sperstad et al., 2009), or SK84 from *Drosophila virilis* that appears to be only active against Gram-positive bacteria (Lu and Chen, 2010). Armadillidins constitute now a new exception within the glycine-rich AMPs family in terms of antimicrobial activity. We must notice that a few microbial strains were tested to establish antimicrobial spectra except for shepherins, coleoptericin, and armadillidins so published results should be interpreted with caution. Moreover, no hemolytic activity was detected against human red blood cells for armadillidin H, as also reported for diptericin A, leptoglycin and SK84 (**Table 3**). The fact that this peptide displayed strong antimicrobial activity but no hemolysis underlines its therapeutic potential even if further toxicity experiments are needed.

**Table 3:** Parameters comparison among some glycine-rich antimicrobial peptides.

Name	Organism	Length	Glycine content (%)	pI	Sensitive strains	^†^MIC (μM)	Hemolytic activity	Reference
Acanthoscurrin 1 and 2	*Acanthoscurria gomesiana*	130 and 132 aa	72.3 and 72.7	10	*C. albicans* MDM8 *E. coli* SBS363	2.3 5.6	^#^ND	Lorenzini et al., 2003
Armadillidin H and Q	*Armadillidium vulgare*	53 aa	47.2	12.01	Gram+, Gram- and filamentous fungi	See **Tables 1** and **2**	No	This work
Coleoptericin	*Zophobas atratus*	74 aa	18	10.9	*E. coli* D22, D31	^#^ND	^#^ND	Bulet et al., 1991
Ctenidin 1, 2, and 3	*Cupiennius salei*	119, 109 and 120 aa	71.4, 70.6, and 71.6	9.6	*E. coli* ATCC 25922	5	^#^ND	Baumann et al., 2010
Diptericin A	*Phormia terranovae*	82 aa	17.9	8.26	*E. coli* D31 *E. herbicola* T *E. carotovora* 113 *S. dispar* P15 *K. pneumoniae* UNF 5023 *X. nematophilus* Xn21	^#^ND	No	Dimarcq et al., 1988; Keppi et al., 1989
Holotricin 3	*Holotrichia diomphalia*	84 aa	64.1	7.41	*C. albicans* 36232	^#^ND	^#^ND	Lee et al., 1995
Hyastatin	*Hyas araneus*	115 aa	27.2	9.84	*C. glutamicum* ATCC 13032 *C. albicans* ATCC 10231 *E. coli* ATCC 25922 *S. cerevisiae*	^∗^0.4 ^∗^6.3–12.5 ^∗^12.5 ^∗^12.5	^#^ND	Sperstad et al., 2009
Leptoglycin	*Leptodactylus pentadactylus*	22 aa	59.1	5.52	*C. freundii* ATCC 8090 *E. coli* ATCC 28922 *P. aeruginosa* ATCC 9027	75 50 8	No	Sousa et al., 2009
SK84	*Drosophila virilis*	84 aa	15.5	6.57	*B. subtilis* ATCC 9372 *B. thuringiensis* ATCC 1041 *S. aureus* ATCC 6538	4 8 8	No	Lu and Chen, 2010
Shepherin I and II	*Capsella bursa-pastoris*	28 and 38 aa	67.9 and 65.8	7.28	^Δ^*C. albicans* ATCC 90028 *C. albicans* HU 168 *C. albicans* MDM8 *C. parapsilosis* ATCC 22019 *C. krusei* ATCC 6258 *C. tropicalis* Squibb 1600 *S. cerevisiae* ATCC 2601	25 25 12.5 6.25 50 1.56 12.5	^#^ND	Park et al., 2000; Remuzgo et al., 2014

*^†^MIC is defined as the lowest concentration of peptide at which the growth is 100% inhibited.*

*^∗^In this paper, MIC was defined as the lowest concentration of peptide causing >50% growth inhibition.*

*^#^Not determined.*

*^Δ^More microbial strains were tested and found sensitive to shepherins. However, they are not listed in the table because authors only provided IC_50_ values.*

The wide antimicrobial spectrum of armadillidins raises the question of how these peptides act on various cells types. Usually, cationic AMPs are able to interact with membranes, leading to a perturbation of the lipid bilayer physical integrity, like membrane thinning and formation of transient pores that could induce cell permeabilization (Li et al., 2012). Peptides could also be translocated across membranes, bringing them in contact with internal targets (Brogden, 2005). Few peptides, like for example the *Apis mellifera* apidaecin, was reported to require a bacterial receptor as the activity of all-D and all-L enantiomers are different (Casteels and Tempst, 1994). First of all, armadillidin H displays a bactericidal effect on both Gram-positive and Gram-negative bacteria and we highlighted that this killing activity occurred very quickly, certainly by a mechanism that does not use signaling pathways but involves more probably membrane permeabilization. With a calculated pI of 12.01, armadillidin H has a positive net charge of +7 at physiological pH, which is consistent with a membranolytic activity (Hancock and Sahl, 2006; Li et al., 2012). Moreover, all of the *P. syringae* cells lost their viability within 30 s while it takes 5 min to achieve the same result for *B. megaterium*. We could hypothesize that specific steps possibly occur to induce bacterial killing and in the case of *P. syringae*, armadillidin H kills bacteria so quickly that it becomes technically challenging to characterize the steps preceding cell death, if there are any. Additionally, we showed that the peptide induced cell membrane damages by a microscopic approach using both scanning and transmission electron microscopy. Indeed, ultrastructure of microbial cells was deeply impacted as many observed cells had lost their shape and membrane integrity, i.e., membrane wrinkling and bubbling. Microscopic analyses of treated *B. megaterium* cells seemed to be in agreement with apparent steps in the action of armadillidin H as different subcellular organization were observed. Also, we showed that permeabilization perfectly correlates with a decrease of cells viability for each tested microorganism. Taken together, our findings indicate that armadillidin H is a membrane active peptide that rapidly induces perturbation of the lipid bilayer and membrane permeabilization thereby provoking, directly or not, cell death. Such biological effects were already described for many AMPs like for example the ovine cathelicidin SMAP29 on *P. aeruginosa* PAO1 cells (Kalfa et al., 2001) or the *Galleria mellonella* defensin on *Legionella dumoffii* cells (Palusinska-Szysz et al., 2012) but never, to our knowledge, for glycine-rich AMPs. Only SK84 was reported to destroy cells membranes but of human leukemia THP-1 cells and no data were given for bacteria excepted MICs (Lu and Chen, 2010).

As underlined by many authors, almost nothing is known about the secondary structure of glycine-rich peptides. To date, only experiments were carried out on shepherin Ia, the carboxyamidated analog of shepherin I, produced by chemical synthesis (Remuzgo et al., 2014). Authors failed to determine the shepherin Ia structure by NMR spectroscopy. However, they observed CD spectra similar to those of cyclic AMPs containing disulfide bridges such as tachyplesin (Rao, 1999), as well as the CD spectra of muscarinic toxin 7, which has a three-finger fold structure comprising five β-strands forming a twisted β-sheet (Fruchart-Gaillard et al., 2012). Concerning armadillidin H, CD spectra recorded with or without TFE are totally superimposable and no additional information was obtained by NMR. Those results emphasize that the peptide do not show any propensity to form secondary structures, in our conditions, even if the media mimics membrane environment.

In summary, the two armadillidin isoforms, armadillidin H and armadillidin Q, are glycine-rich peptides with a similar broad spectrum antimicrobial activity. They act in the micromolar range against Gram-negative and Gram-positive bacteria, filamentous fungi but were totally inactive against yeasts. Armadillidin H was shown to be membrane active against *P. syringae, B. megaterium*, and *A. brassissicola*, leading to deep changes in cell morphology. This damaging activity correlates to a rapid decrease of cell viability, leading to highly blebbed cells with subsequent leakage of their cytoplasmic contents. No secondary structure could be defined in this study even in a membrane mimicking environment. However, the GGGF(H/N)(R/S) repeated motif encountered in armadillidins could be critical for structure and activities, similarly to the GGH motif of shepherins (Park et al., 2000). Further studies will thus be necessary to better understand structure-function relationship and other properties like the apparent lack of cytotoxicity (no hemolytic activity). Therefore, armadillidins represent interesting candidates to gain insight into the biology of glycine-rich AMPs.

## Author Contributions

JV, PC-T, SC, PG, DB, J-MB, and CB-V conceived and designed the study. JV, PC-T, M-HR, CL, SD, CN, SC, BM, and J-MB carried out the experiments. JV, PC-T, CL, J-MB, and CB-V wrote the paper.

## Conflict of Interest Statement

The authors declare that the research was conducted in the absence of any commercial or financial relationships that could be construed as a potential conflict of interest.

## Footnotes

1 http://blast.ncbi.nlm.nih.gov/
