# Supplementary material for: Armadillidin H, a Glycine-Rich Peptide from the Terrestrial Crustacean Armadillidium vulgare, Displays an Unexpected Wide Antimicrobial Spectrum with Membranolytic Activity
Source: Front Microbiol. 2016 Sep 22;7:1484. doi: 10.3389/fmicb.2016.01484 (PMC5031766; doi:10.3389/fmicb.2016.01484)
Supplement: Supplementary file 1 [file Image_1.PDF]

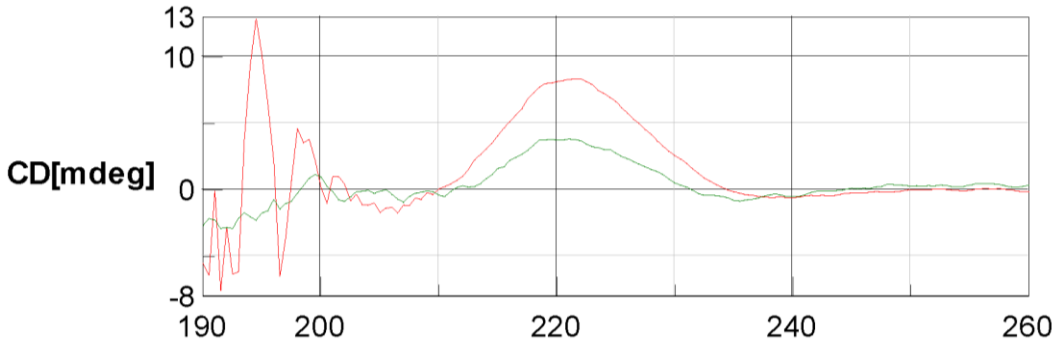

Supp data: Superimposition of CD spectra or armadillidin H recorded without TFE (red) and after the addition of 50% of TFE (green).

NOESY = Nuclear Overhauser Effect SpectroscopY, TOCSY = TOtal Correlation SpectroscopY, HSQC Heteronuclear Single Quantum Coherence
